# Association of Total Fluid Intake and Output with Duration of Hospital Stay in Patients with Acute Pancreatitis

**DOI:** 10.1155/2018/7614381

**Published:** 2018-05-02

**Authors:** Andree H. Koop, Fernando F. Stancampiano, Jillian Jackson, April Henry, Jennifer Horsley-Silva, Rahul Pannala, Michael G. Heckman, Nancy N. Diehl, William C. Palmer

**Affiliations:** ^1^Department of Internal Medicine, Mayo Clinic, Jacksonville, FL, USA; ^2^CRISP Student Program, Mayo Clinic, Jacksonville, FL, USA; ^3^Department of Gastroenterology and Hepatology, Mayo Clinic, Scottsdale, AZ, USA; ^4^Division of Biomedical Statistics and Informatics, Mayo Clinic, Jacksonville, FL, USA; ^5^Department of Gastroenterology and Hepatology, Mayo Clinic, Jacksonville, FL, USA

## Abstract

**Background/Aims:**

The aim of this study was to evaluate the association of fluid balance with outcomes in patients hospitalized with acute pancreatitis (AP).

**Methods:**

This was a retrospective study of patients hospitalized between May 2008 and June 2016 with AP and a clinical order for strict recording of intake and output. Data collected included various types of fluid intake and output at 24 and 48 hours after admission. The primary outcome was length of stay (LOS). Analysis was performed using single-variable and multivariable negative binomial regression models.

**Results:**

Of 1256 patients hospitalized for AP during the study period, only 71 patients (5.6%) had a clinical order for strict recording of intake and output. Increased urine output was associated with a decreased LOS at 24 and 48 hours in univariable analysis. An increasingly positive fluid balance (total intake minus urine output) at 24 hours was associated with a longer LOS in multivariable analysis.

**Conclusions:**

Few patients hospitalized for AP had a documented order for strict monitoring of fluid intake and output, despite the importance of monitoring fluid balance in these patients. Our study suggests an association between urine output and fluid balance with LOS in AP.

## 1. Introduction

Acute pancreatitis (AP), an inflammatory disease of the pancreas, is one of the most common gastrointestinal disorders and has significant morbidity, mortality, and healthcare costs [1]. In 2012, it accounted for nearly 280,000 hospitalizations in the United States with a median length of stay of four days and a cost of nearly 2.6 billion dollars [2]. Despite advances in medical therapy, the incidence of AP is increasing with an overall mortality approaching 5%, and as high as 47% in patients with multiorgan failure [3, 4]. AP is generally diagnosed on the basis of two of the following three features: abdominal pain, serum lipase or amylase greater than four times the upper limit of normalcy, and characteristic findings on abdominal imaging [5, 6].

Despite advances in the knowledge and pathophysiology of AP, there are no targeted pharmacologic therapies and the cornerstone of management is intravenous fluid resuscitation [7, 8]. Recent studies have illustrated the importance of fluid resuscitation in AP with improved morbidity and mortality [9–11]. Urine output and total fluid balance are important clinical endpoints to monitor, with increased fluid sequestration associated with worse outcomes in AP [12, 13]. The primary aim of this study was to evaluate the associations of fluid balance and strictly monitored fluid intake and output (total and specific types) at 24 and 48 hours of hospital admission with outcomes in AP.

## 2. Methods

### 2.1. Study Design and Setting

We conducted a two-center retrospective analysis of patients admitted to Mayo Clinic Florida, a 300-bed academic tertiary referral center, and Mayo Clinic Arizona, a 260-bed academic tertiary referral center. The study protocol was approved by the Mayo Clinic Institutional Review Board.

### 2.2. Study Patients and Data

The electronic medical records at Mayo Clinic Florida and Mayo Clinic Arizona were searched for patients hospitalized between May 1, 2008, and June 1, 2016, with a serum lipase ≥ 240 *μ*/L and a documented clinical order for strict monitoring of intake and output written within the first 12 hours of hospitalization. Patients ≤ 18 years old were excluded. A chart review was performed to confirm the diagnosis and the etiology of acute pancreatitis in each patient. In addition to a serum lipase ≥ 240 *μ*/L, patients required abdominal pain or clinical imaging findings consistent with AP to confirm the diagnosis. All included patients were required to have AP listed as a discharge diagnosis. Patients without a confirmed diagnosis of AP were excluded. Information was collected regarding baseline patient characteristics and laboratory values, clinical course, and various types of fluid intake and output at 24 and 48 hours after admission. For fluid output at 24 and 48 hours after admission, urine output, other fluid output (i.e., not urine), and total fluid output were all measured. Additionally, as measures of fluid balance, we calculated the differences and ratios between total fluid intake and total fluid output and also the differences and ratios between total fluid intake and urine output, both at 24 and 48 hours after admission.

### 2.3. Study Outcomes

The primary study outcome was LOS. Due to the low sample size of our study, there was no sufficient power to analyze other outcomes. When evaluating associations of total fluid intake and urine output at 24 hours, the outcome that was utilized was LOS after that 24-hour time point. Similarly, when examining associations of total fluid intake and urine output at 48 hours after hospital admission, the outcome that was utilized was LOS after that 48-hour time point. Six patients were hospitalized for less than two days and were excluded from all analyses assessing associations of fluid intake and output at 48 hours after hospital admission with LOS.

### 2.4. Statistical Analysis

Continuous variables were summarized with the sample median and range. Categorical variables were summarized with number and percentage of patients. Associations of fluid intake, fluid output, and fluid balance (i.e., differences and ratios between fluid intake and fluid output) at 24 and 48 hours after hospital admission with LOS after those two time points were evaluated using single-variable (i.e., unadjusted) and multivariable negative binomial regression models [14]. Multivariable models were adjusted for the variables showing the strongest association (i.e., lowest *P* value) with LOS while including no more than one variable in a given model for every 10 patients utilized in the analysis per recommended guidelines [15]. In the event that two of the variables showing the strongest associations with LOS were very highly correlated and therefore provided redundant information (which was the case for creatinine and BUN), only one of these two variables (i.e., the variable with the lowest association *P* value) was adjusted for in the multivariable model. Multiplicative effects on mean LOS were estimated along with 95% confidence intervals.

For total fluid intake, urine output, total fluid output, and differences/ratios between fluid intake and fluid output at 24 and 48 hours after hospital admission, these variables were assessed as continuous variables in negative binomial regression analysis. Specific types of fluid intake were all assessed as binary categorical variables based on the sample median (≤median versus >median) in regression analysis due to the high concentration of zero values for most of the specific types of fluid intake. Only specific types of fluid intake for which there were more than five patients with a value greater than zero were assessed for association with LOS. All statistical tests were two sided, and *P* values of 0.05 or lower were considered statistically significant. All statistical analyses were performed using SAS (version 9.2; SAS Institute Inc., Cary, North Carolina) and R Statistical Software (version 3.1.1; R Foundation for Statistical Computing, Vienna, Austria).

## 3. Results

**Figure 1:**
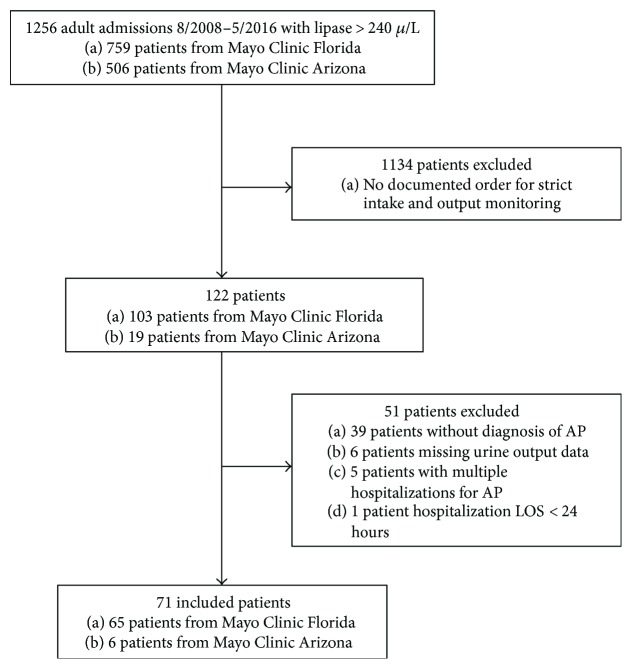
Study inclusion criteria.

The two study sites had a total of 1256 patients admitted with acute pancreatitis during the study period; however, only 71 (5.6%) of those patients met the inclusion criteria, specifically a physician's order for strict intake and output monitoring ensuring the accurate measurement of these endpoints (Figure 1). A summary of baseline patient characteristics, hospitalization information, and posthospitalization outcomes is provided in Table 1. Median age at admission was 59 years (range: 21–93 years) and 29 patients (41%) were female. Primary etiology was alcohol abuse (36%), biliary disease (32%), or other (32%). BISAP score was 0 in 22 patients (31%), 1 in 19 patients (27%), and 2 or higher in 30 patients (42%). A total of 17 patients (24%) experienced a complication during hospitalization, including intra-abdominal infection (*N* = 3), need for surgery (*N* = 2), need for ERCP (*N* = 5), and pancreatic necrosis on CT/MRI (*N* = 10). Admission to the ICU (9%), vasopressor use (13%), mechanical ventilation (4%), hemodialysis/CRRT (6%), death within 30 days of discharge (3%), and readmission within 30 days of discharge (13%) were all fairly rare. The median LOS was 4 days (range: 1–37 days).

Fluid intake and output information at 24 and 48 hours after hospital admission is summarized in Table 2. The median fluid intake at 24 and 48 hours after admission was 5108 mL (range: 160–15,208 mL) and 8120 mL (range: 532–16,972 mL), respectively. Normal saline and lactate ringer were the fluids given most often. Urine output was a median of 1250 mL (range: 0–4250 mL) at 24 hours after hospital admission and 2975 mL (range: 0–8625 mL) at 48 hours after hospital admission.

To better understand variables that may confound associations of fluid intake and output at 24 and 48 hours after hospital admission with LOS, we first examined associations of baseline characteristics and LOS; these results are shown in Table 3. The variables that showed the strongest degree of association with LOS were systolic blood pressure (*P* < 0.0001), BISAP score (*P* < 0.0001), bicarbonate (*P* < 0.0001), creatinine (*P* < 0.0001), BUN (*P* < 0.0001), heart rate (*P* = 0.0005), and hematocrit (*P* = 0.0005). Due to the fact that creatinine and BUN were very highly correlated (Spearman's *r*: 0.79, *P* < 0.0001), we only adjusted for the latter due to its stronger association with LOS in our subsequent multivariable analysis evaluating associations of fluid intake and output with LOS.

In the evaluation of the primary and secondary study aims, Table 4 displays the associations of fluid intake and output at 24 and 48 hours following hospital admission with subsequent LOS. In single-variable analysis, variables that were associated with a significantly longer LOS were a greater “other” intake at 24 hours (*P* = 0.0002), a greater albumin intake at 48 hours (*P* = 0.0004), a greater blood product intake at 48 hours (*P* = 0.002), a greater D5W or D10W intake at 48 hours (*P* = 0.003), a greater other intake at 48 hours (*P* < 0.0001), and a greater ratio of total fluid intake/urine output at both 24 hours (*P* = 0.030) and 48 hours (*P* = 0.005). Variables that were associated with a significantly shorter LOS in single-variable analysis included a greater oral intake at 24 hours (*P* = 0.035), a greater urine output at 24 hours (*P* = 0.030), and a greater urine output at 48 hours (*P* = 0.004).

In multivariable analysis adjusting for the potential confounding influences of respiratory rate, systolic blood pressure, BISAP score, hematocrit, bicarbonate, and BUN, many of these associations weakened noticeably. The only associations that remained statistically significant independently of the aforementioned potential confounding variables were those between a greater other intake at 24 hours (*P* = 0.007) and 48 hours (*P* < 0.0001) and a longer LOS and the association between a greater albumin intake at 48 hours and a longer LOS (*P* = 0.011). Additionally, there was a significant association between a greater difference between total fluid intake and urine output at 24 hours and a longer LOS in multivariable analysis (*P* = 0.048) that did not quite reach statistical significance in single-variable analysis (*P* = 0.064).

## 4. Discussion

The two most important conclusions from our study of patients hospitalized with acute pancreatitis were that a greater urine output at 24 hours and 48 hours of hospital admission may be associated with a shorter LOS and also that an increasingly positive fluid balance at 24 hours appears to be associated with a longer LOS, independently of other variables such as BISAP score. The first conclusion is based on our finding that increased urine output at 24 and 48 hours was associated with a decreased length of stay following those two time points in an unadjusted analysis. These findings were not significant in multivariable analysis, which was not surprising given that some of the variables adjusted for, including systolic blood pressure, BUN, and bicarbonate, are highly related to urine output. Our second conclusion is based on our multivariable model demonstrating an association between increasingly positive fluid balance (total oral and IV intake minus urine output) and a longer LOS. Although we recognize the relatively small size of our patient cohort and the corresponding need for validation of our findings, this data supports the concept that strict urine output and fluid balance are important clinical endpoints to monitor in acute pancreatitis.

Surprisingly, only 71 of 1256 patients (5.6%) with AP had a physician's order for strict intake and output monitoring which was the primary limitation of the study sample size. At the two study centers, the clinical order for strict intake and output monitoring prompts the collection and measurement of all oral intake and urine output, ensuring the accurate documentation of fluid balance on hospitalized patients. Without this order, accurate measurement and documentation of fluid balance are not guaranteed. Given the importance of fluid resuscitation and urine output monitoring in the management of AP, we argue that this order should be placed on all hospitalized patients with AP. The order is placed at the discretion and clinical judgement of the treating physician, and it is possible that the included patients with this order had a more severe disease course. The local practice of using this order could explain differences in the sample size from the two study sites (65 versus 6 patients).

Another important finding of this study was the association between other intake and albumin intake with a longer hospital stay. These findings were significant in both single-variable and multivariable analyses. Other intake primarily included the infusion of medications such as anesthetics, paralytics, antibiotics, and opiates in patients with a more complicated hospital course. In single-variable analysis, the administration of blood products, D5W or D10W, was also associated with a more prolonged stay, although this association was no longer significant in multivariable analysis. Together, these findings support the conclusion that patients requiring additional intake other than intravenous fluids have a longer length of hospital stay, likely secondary to medical comorbidities and/or a more complicated disease course.

Urine output allows for an assessment of intravascular volume repletion [8, 16–19]. Fluid resuscitation is important in acute pancreatitis to perfuse the pancreatic microcirculation and resolve hypovolemia. The microcirculation is disrupted in AP, and hypoperfusion has been suggested as a risk factor for the development of severe AP and pancreatic necrosis [20, 21]. Additionally, AP causes the release of proinflammatory mediators and cytokines leading to vasodilation, increased vascular permeability, and the systemic inflammatory response which can ultimately cause intravascular volume depletion, hypotension, and shock [7, 8]. Patients with AP are at risk for hypovolemia as they frequently present with vomiting, decreased oral intake, and diaphoresis [8]. The monitoring of urine volumes in patients with AP has always been logical and intuitive, but further research is needed to identify thresholds of urine output associated with greater morbidity and mortality in AP. The results of an association between fluid intake and urine output with LOS may imply that a fluid balance approaching net even versus a positive balance may be preferred, although the degree to which this should occur is unknown.

Few studies have evaluated the length of hospital stay in patients with AP. In 2009, the estimated median length of hospital stay for patients with AP was 4 days, which is identical to the one in our study [1]. In a prospective study of 403 patients at two institutions, increased fluid sequestration at 48 hours after hospital admission was significantly associated with a longer hospital stay as well as higher rates of acute fluid collections, pancreatic necrosis, and persistent organ failure. The median level of fluid sequestration was 3.2 L at 48 hours, which is similar to that of our study although we did not account for insensible fluid losses [12]. A retrospective study of 227 patients with a median fluid sequestration of 4.2 L at 48 hours found no significant association with the length of stay with a *P* value of 0.09 [13].

Two other groups reported no association between fluid resuscitation and LOS. A retrospective study of early versus late fluid resuscitation in 45 patients with severe AP found no difference in LOS between the two groups [9]. In a randomized controlled trial of 40 patients comparing goal-directed to standard fluid resuscitation with normal saline and lactated Ringer's solution, there was no difference in LOS amongst the four treatment arms [22]. Additionally, few studies have evaluated the association of urine output with outcomes in AP; the majority focused on fluid resuscitation [23, 24].

Our study did not find an association between total fluid intake and length of stay. In a retrospective study of early versus late fluid resuscitation of 434 patients with mild and severe AP, early intervention correlated with a significantly decreased LOS (8 versus 11 days) [10]. These findings were more pronounced in patients with interstitial pancreatitis, and LOS was not significantly higher in patients with severe AP [10].

Colloids have not been as well studied as crystalloids in the management of acute pancreatitis in humans [8]. Our study found that the use of albumin was associated with a longer hospital stay, likely reflecting the severity of illness in these patients. A Cochran analysis found that fluid resuscitation with hydroxyethyl starch may increase mortality in critically ill patients. Further studies are needed to evaluate the benefit of these colloids in acute pancreatitis [25].

In addition to its retrospective design, our study has other limitations, the main one being its relatively small sample size, which results in a lack of power to detect associations. The possibility of a type II error (i.e., a false-negative finding) is important as we cannot conclude that there is no true association simply based on the lack of a statistically significant *P* value. The low sample size limited our ability to evaluate the associations between total fluid intake and output and other outcomes of acute pancreatitis including local complications and mortality. We did not closely account for other factors in the management of AP which could have led to confounding, such as early enteral feeding or antibiotic use which was a component of other fluid output. Finally, other than BISAP score calculations, we did not further stratify patients by the severity of AP. Since patients with mild acute pancreatitis generally have a benign and uncomplicated clinical course compared to those with severe disease, it is possible that the two groups may have had different clinical outcomes with regard to fluid intake and output.

## 5. Conclusion

This study evaluated the association between total fluid intake and output with LOS as the primary outcome in patients with AP. Few patients hospitalized for AP had an order for strict monitoring of intake and output, which is important to ensure the accurate documentation of these endpoints. There were significant associations between urine output, fluid balance, and LOS. These findings suggest that patients hospitalized with AP should have close monitoring of their fluid balance and urine output as it has implications for clinical outcomes. Validation in larger patient cohorts and randomized controlled trials will be important.

## Figures and Tables

**Table 1:** Baseline patient characteristics, clinical course, and outcomes.

Variable	Summary (*N* = 71)
Baseline characteristics	
Age (years)	59 (21, 93)
Sex (female)	29 (40.8%)
Primary etiology	
ETOH	19 (35.8%)
Biliary	17 (32.1%)
Other	17 (32.1%)
Antihypertensive medications at the time of hospital admission	
Diuretic	6 (8.5%)
Other antihypertensive medication	17 (23.9%)
BMI	27.5 (18.0, 48.2)
Heart rate	91 (53, 147)
Respiratory rate	18 (8, 51)
Systolic blood pressure	133 (75, 218)
Diastolic blood pressure	78 (36, 133)
Oxygen saturation	98 (81, 100)
BISAP score	
0	22 (31.0%)
1	19 (26.8%)
2	27 (38.0%)
3	2 (2.8%)
4	1 (1.4%)
Hematocrit	38.4 (24.7, 51.5)
Hemoglobin	13.2 (8.3, 18.5)
Sodium	137 (117, 146)
Potassium	4 (2.8, 11.7)
Calcium	8.6 (5.3, 10.9)
Bicarbonate	24 (6, 32)
Creatinine	0.9 (0.5, 14.9)
BUN	16 (5, 127)
Triglycerides	132 (42, 3173)
Clinical course and outcomes	
Complications	
Any complication	17 (23.9%)
Intra-abdominal infection	3 (17.6%)
Need for surgery	2 (11.8%)
Need for ERCP	5 (29.4%)
Pancreatic necrosis on CT/MRI	10 (58.8%)
Admitted to ICU	6 (8.5%)
Length of hospital stay	4 (1, 37)
Vasopressor use during hospitalization	9 (12.7%)
Dopamine	0 (0.0%)
Dobutamine	1 (1.4%)
Epinephrine	1 (1.4%)
Norepinephrine	3 (4.2%)
Vasopressin	1 (1.4%)
Phenylephrine	7 (9.9%)
Mechanical ventilation during hospitalization	3 (4.2%)
Hemodialysis/CRRT	4 (5.6%)
Death within 30 days of hospital discharge	2 (2.8%)
Readmission within 30 days of hospital discharge	9 (12.7%)

Continuous variables were summarized with the sample median (minimum, maximum). Information was unavailable regarding primary etiology (*N* = 18), calcium (*N* = 2), and triglycerides (*N* = 30).

**Table 2:** Fluid intake and output information at 24 and 48 hours after hospital admission.

Variable	Summary (*N* = 71)
Fluid intake at 24 hours following admission (mL)	
Normal saline	2000 (0, 7869)
0.5 normal saline	0 (0, 743)
Lactate ringer	250 (0, 6695)
Albumin	0 (0, 2999)
Blood products	0 (0, 1275)
Oral intake	0 (0, 1250)
Tube feeding	0 (0, 688)
D5W or D10W	0 (0, 893)
Sodium bicarbonate	0 (0, 1400)
TPN/PPN	0 (0, 1102)
Other intake	2 (0, 1652)
Total intake	5108 (160, 15,208)
Fluid intake at 48 hours following admission (mL)	
Normal saline	2700 (0, 11,846)
0.5 normal saline	0 (0, 2400)
Lactate ringer	1000 (0, 10,450)
Albumin	0 (0, 3394)
Blood products	0 (0, 1275)
Oral intake	480 (0, 2200)
Tube feeding	0 (0, 1756)
D5W or D10W	0 (0, 1887)
Sodium bicarbonate	0 (0, 1400)
TPN/PPN	0 (0, 1862)
Other intake	50 (0, 1850)
Total intake	8120 (532, 16,972)
Fluid output at 24 hours following admission (mL)	
Urine output	1250 (0, 4250)
Other output	0 (0, 5750)
Total fluid output	1450 (0, 6265)
Fluid output at 48 hours following admission (mL)
Urine output	2975 (0, 8625)
Other output	0 (0, 5750)
Total fluid output	3420 (0, 8625)
Fluid balance at 24 hours following admission	
Total intake minus total output	3242 (−3779, 12,908)
Total intake minus urine output	3270 (−800, 12,908)
Ratio of total intake/total output	3.24 (0.38, 5129)
Ratio of total intake/urine output	3.84 (0.60, 5129)
Fluid balance at 48 hours following admission	
Total intake minus total output	4479 (−2832, 13,562)
Total intake minus urine output	4542 (−1957, 13,562)
Ratio of total intake/total output	2.37 (0.58, 7523)
Ratio of total intake/urine output	2.68 (0.60, 7523)

Continuous variables were summarized with the sample median (minimum, maximum).

**Table 3:** Associations between baseline patient characteristics and length of hospital stay.

Variable	Multiplicative effect on mean LOS (95% CI)	*P* value
Age at hospital admission (10-year increase)	1.00 (0.98, 1.01)	0.61
Sex (female)	1.29 (0.79, 2.11)	0.30
Primary etiology	Test of overall difference: *P* = 0.012	
ETOH	1.00 (reference)	N/A
Biliary	0.95 (0.52, 1.73)	0.88
Other	2.07 (1.17, 3.66)	0.012
Any antihypertensive medication at the time of hospital admission	0.85 (0.49, 1.48)	0.57
BMI (5-unit increase)	1.17 (0.93, 1.47)	0.17
Heart rate (10-unit increase)	1.19 (1.08, 1.32)	0.0005
Respiratory rate (10-unit increase)	1.91 (1.36, 2.69)	0.0002
Systolic blood pressure (10-unit increase)	0.86 (0.79, 0.92)	<0.0001
Diastolic blood pressure (10-unit increase)	0.86 (0.76, 0.96)	0.0102
Oxygen saturation (5-unit increase)	0.98 (0.66, 1.44)	0.92
BISAP score	Test of overall difference: *P* < 0.0001	
0	1.00 (reference)	N/A
1	1.42 (0.77, 2.61)	0.26
2–4	3.11 (1.83, 5.30)	<0.0001
Hematocrit (10-unit increase)	0.53 (0.37, 0.76)	0.0005
Hemoglobin (1-unit increase)	0.87 (0.79, 0.95)	0.0028
Sodium (5-unit increase)	0.69 (0.54, 0.88)	0.0032
Potassium (1-unit increase)	1.13 (0.89, 1.43)	0.32
Calcium (1-unit increase)	0.77 (0.65, 0.92)	0.0040
Bicarbonate (5-unit increase)	0.55 (0.42, 0.72)	<0.0001
Creatinine (doubling)	1.73 (1.36, 2.19)	<0.0001
BUN (doubling)	1.76 (1.41, 2.21)	<0.0001
Triglycerides (doubling)	1.25 (1.04, 1.49)	0.0177

LOS = length of stay; CI = confidence interval. Multiplicative effects, 95% CIs, and *P* values result from single-variable (i.e., unadjusted) negative binomial regression models.

**Table 4:** Associations of total fluid intake and urine output at 24 and 48 hours after hospital admission with length of hospital stay.

Variable	*N*	Single-variable analysis		Multivariable analysis	
		Multiplicative effect on mean LOS (95% CI)	*P* value	Multiplicative effect on mean LOS (95% CI)	*P* value
At 24 hours following hospital admission					
Normal saline (>2000 mL)	71	1.14 (0.70, 1.85)	0.60	0.85 (0.57, 1.25)	0.41
Lactate ringer (>250 mL)	71	0.71 (0.44, 1.15)	0.16	1.22 (0.83, 1.79)	0.31
Oral intake (>0 mL)	71	0.60 (0.37, 0.96)	0.035	0.83 (0.56, 1.21)	0.33
Other intake (>2 mL)	71	2.38 (1.51, 3.75)	0.0002	1.72 (1.16, 2.54)	0.007
Total fluid intake (per 1000 mL increase)	71	1.04 (0.95, 1.14)	0.38	1.06 (0.99, 1.13)	0.12
Urine output (per 500 mL increase)	71	0.89 (0.80, 0.99)	0.030	0.98 (0.89, 1.07)	0.64
Total fluid output (per 500 mL increase)	71	1.06 (0.98, 1.15)	0.17	1.03 (0.96, 1.10)	0.47
Total intake minus total output (per 2500 mL increase)	71	1.01 (0.84, 1.21)	0.93	1.11 (0.94, 1.32)	0.22
Total intake minus urine output (per 2500 mL increase)	71	1.27 (0.99, 1.63)	0.064	1.21 (1.00, 1.47)	0.048
Ratio of total intake/total output (doubling)	71	0.98 (0.89, 1.07)	0.65	1.00 (0.93, 1.08)	0.91
Ratio of total intake/urine output (doubling)	71	1.13 (1.01, 1.27)	0.030	1.02 (0.95, 1.10)	0.59
At 48 hours following hospital admission					
Normal saline (>2700 mL)	65	0.99 (0.54, 1.80)	0.96	0.81 (0.50, 1.32)	0.40
Lactate ringer (>1000 mL)	65	0.67 (0.37, 1.22)	0.19	1.40 (0.85, 2.33)	0.19
Albumin (>0 mL)	65	4.75 (2.01, 11.22)	0.0004	2.57 (1.25, 5.31)	0.011
Blood products (>0 mL)	65	4.21 (1.73, 10.24)	0.002	1.98 (0.89, 4.44)	0.096
Oral intake (>480 mL)	65	0.56 (0.31, 1.02)	0.057	0.82 (0.50, 1.36)	0.45
D5W or D10W (>0 mL)	65	3.90 (1.58, 9.63)	0.003	2.06 (0.99, 4.28)	0.054
Other intake (>50 mL)	65	4.52 (2.66, 7.69)	<0.0001	2.95 (1.95, 4.47)	<0.0001
Total fluid intake (per 1000 mL increase)	65	0.99 (0.91, 1.07)	0.76	1.02 (0.95, 1.10)	0.54
Urine output (per 500 mL increase)	65	0.88 (0.83, 0.95)	0.004	0.96 (0.90, 1.02)	0.18
Total fluid output (per 500 mL increase)	65	1.02 (0.93, 1.11)	0.67	1.00 (0.94, 1.06)	0.88
Total intake minus total output (per 2500 mL increase)	65	0.95 (0.78, 1.16)	0.63	1.06 (0.89, 1.26)	0.51
Total intake minus urine output (per 2500 mL increase)	65	1.25 (0.98, 1.58)	0.071	1.14 (0.95, 1.38)	0.16
Ratio of total intake/total output (doubling)	65	0.91 (0.75, 1.09)	0.30	0.98 (0.83, 1.14)	0.75
Ratio of total intake/urine output (doubling)	65	1.24 (1.07, 1.44)	0.005	1.04 (0.94, 1.16)	0.45

LOS = length of stay; CI = confidence interval. Multiplicative effects, 95% CIs, and *P* values result from negative binomial regression models. For analysis involving total fluid intake and urine output at 24 hours following hospital admission, multivariable models were adjusted for respiratory rate, systolic blood pressure, BISAP score, hematocrit, bicarbonate, and BUN. For analysis involving total fluid intake and urine output at 48 hours following hospital admission, multivariable models were adjusted for respiratory rate, systolic blood pressure, BISAP score, bicarbonate, and BUN. One fewer variable was adjusted for in the multivariable models involving total fluid intake and urine output at 48 hours following hospital admission due to the smaller sample size utilized in that analysis. For measures involving total fluid intake, urine output, and total fluid output at 24 and 48 hours after hospital admission, these were assessed as continuous variables in negative binomial regression analysis. Specific types of fluid intake were all assessed as binary categorical variables based on the sample median (≤median versus >median) in negative binomial regression analysis due to the high concentration of zero values for most of the specific types of fluid intake. Only specific types of fluid intake for which there were more than five patients with a value greater than zero were assessed for the association with LOS.
